# Optimising conditions for bioethanol production from rice husk and rice straw: effects of pre-treatment on liquor composition and fermentation inhibitors

**DOI:** 10.1186/s13068-018-1062-7

**Published:** 2018-03-09

**Authors:** Jia Wu, Adam Elliston, Gwenaelle Le Gall, Ian J. Colquhoun, Samuel R. A. Collins, Ian P. Wood, Jo Dicks, Ian N. Roberts, Keith W. Waldron

**Affiliations:** 1 The Biorefinery Centre, Quadram Institute Bioscience, Norwich Research Park, Colney, Norwich, NR4 7UA UK; 2 The Analytical Sciences Unit, Quadram Institute Bioscience, Norwich Research Park, Colney, Norwich, NR4 7UA UK; 3 The National Collection of Yeast Cultures, Quadram Institute Bioscience, Norwich Research Park, Colney, Norwich, NR4 7UA UK

**Keywords:** Lignocellulosic biomass, Rice husk, Rice straw, Pre-treatment, Inhibitors, Saccharification, Xylo-oligomers, Fermentation, Bioethanol

## Abstract

**Background:**

Rice straw and husk are globally significant sources of cellulose-rich biomass and there is great interest in converting them to bioethanol. However, rice husk is reportedly much more recalcitrant than rice straw and produces larger quantities of fermentation inhibitors. The aim of this study was to explore the underlying differences between rice straw and rice husk with reference to the composition of the pre-treatment liquors and their impacts on saccharification and fermentation. This has been carried out by developing quantitative NMR screening methods.

**Results:**

Air-dried rice husk and rice straw from the same cultivar were used as substrates. Carbohydrate compositions were similar, whereas lignin contents differed significantly (husk: 35.3% w/w of raw material; straw 22.1% w/w of raw material). Substrates were hydrothermally pre-treated with high-pressure microwave processing across a wide range of severities. 25 compounds were identified from the liquors of both pre-treated rice husk and rice straw. However, the quantities of compounds differed between the two substrates. Fermentation inhibitors such as 5-HMF and 2-FA were highest in husk liquors, and formic acid was higher in straw liquors. At a pre-treatment severity of 3.65, twice as much ethanol was produced from rice straw (14.22% dry weight of substrate) compared with the yield from rice husk (7.55% dry weight of substrate). Above severities of 5, fermentation was inhibited in both straw and husk. In addition to inhibitors, high levels of cellulase-inhibiting xylo-oligomers and xylose were found and at much higher concentrations in rice husk liquor. At low severities, organic acids and related intracellular metabolites were released into the liquor.

**Conclusions:**

Rice husk recalcitrance to saccharification is probably due to the much higher levels of lignin and, from other studies, likely high levels of silica. Therefore, if highly polluting chemical pre-treatments and multi-step biorefining processes are to be avoided, rice husk may need to be improved through selective breeding strategies, although more careful control of pre-treatment may be sufficient to reduce the levels of fermentation inhibitors, e.g. through steam explosion-induced volatilisation. For rice straw, pre-treating at severities of between 3.65 and 4.25 would give a glucose yield of between 37.5 and 40% (w/DW, dry weight of the substrate) close to the theoretical yield of 44.1% w/DW, and an insignificant yield of total inhibitors.

**Electronic supplementary material:**

The online version of this article (10.1186/s13068-018-1062-7) contains supplementary material, which is available to authorized users.

## Background

The energy crisis and how to address it has been long debated, encompassing a wide range of topics from the economic implications of climate change and “peak oil” to the improvements in technologies for producing renewable or low carbon energy. Renewable and low carbon electrical energy is a rapidly developing sector involving nuclear, wind power or photovoltaic technologies [1, 2]. However, the bulk of road vehicles require liquid fuels and this has led to global programmes for producing renewable biofuels that have the potential to be sustainable, and emit minimal levels of greenhouse gases [3, 4].

Of interest are second-generation biofuels such as cellulosic bioethanol. Cellulose is the most abundant source of glucose, and is found in lignocellulosic biomass and wastes including agricultural residues such as forestry residues and pulping wastes, cereal straws, and threshing husks, as well as food processing coproducts such as brewers spent grain [5, 6]. As Rajaram and Varma [7] reported in 1990, there were about 2900 million tonnes of lignocellulosic waste from cereal crops, 160 million tonnes from pulse crops, 14 million tonnes from oilseed crops and 540 million tonnes from plantation crops.

Rice is one of the most widely grown cereal crops, with enormous levels of production in Asian countries leading to an abundance of rice husk and rice straw lignocellulosic wastes [8]. The world annual production of rice husk has been reported as approximately 120 million tonnes [9]. Kim and Dale [10] reported that 667.59 million tonnes rice straw were at that time produced in Asia, and Binod and colleagues [11] calculated that this could theoretically be converted into 281.72 billion litres of ethanol.

However, converting the cellulose and other cell wall sugars to ethanol is highly challenging due to the protective biochemical and structural nature of the lignocellulose [12], which hampers the hydrolysis of the polysaccharides to fermentable monosaccharides [13, 14]. Generically, the conversion processes employed comprise four main steps: hydrothermal pre-treatment, enzymatic or chemical saccharification, fermentation and purification. The aim of pre-treatment is to separate the lignin from the cellulose, reduce the structural barriers created by hemicelluloses, reduce cellulosic crystallinity and thereby improve the accessibility of cellulose to cellulases [15, 16]. The fermentable sugars released can be latterly converted to products by microorganisms such as bacteria and yeasts [17, 18]. Finally, the product of interest can be recovered from the fermentation liquor, for example by distillation. Each of all those steps has a range of options, and the different combinations of those four steps can cause various results.

Previously, we systematically demonstrated that rice straw and rice husk exhibit very different propensities for enzymatic saccharification and fermentation behaviour in response to steam explosion pre-treatment [19]. The aim of this study has been to evaluate in greater depth the differences in the composition of these lignocellulosic materials, and the changes that occur in them during hydrothermal pre-treatments relevant to their biorefining potential, with special reference to the release of potential fermentation inhibitors and related chemicals. This has been achieved by using enclosed hydrothermal pre-treatment conditions to avoid loss of volatile substances that might occur during steam explosion. Furthermore, by using variations of time and temperature, a much higher range of pre-treatment severities have been assessed. Conditions conducive to optimal simultaneous saccharification and fermentation have also been explored.

## Results

### Sugar and lignin analysis of air-dried rice husk and rice straw

Sugar compositions in both rice husk and rice straw comprised rhamnose, fucose, arabinose, xylose, mannose, galactose and glucose (Table 1) and are in keeping with previous studies [9, 20–22]. Uronic acid was not quantified. Cellulose-derived glucose was the most abundant sugar (38.7% in rice straw and 36.8% in rice husk) followed by hemicellulosic xylose (22.9% in rice straw and 19.7% in rice husk). Lignin (corrected for ash; Table 1) was much higher in rice husk (35% w/w) compared with straw (22.1% w/w).

**Table 1:** Sugar and lignin analysis of milled, air-dried rice husk and rice straw

Components	Rice husk (~ %DW)	Rice straw (~ %DW)
Rhamnose	0.23 ± 0.01	0.25 ± 0.01
Fucose	0.05 ± 0.00	0.06 ± 0.01
Arabinose	2.58 ± 0.03	3.94 ± 0.15
Xylose	19.66 ± 0.46	22.93 ± 0.32
Mannose	0.44 ± 0.26	0.30 ± 0.02
Galactose	1.28 ± 0.09	1.40 ± 0.05
Glucose	36.83 ± 0.21	38.66 ± 0.65
Lignin	35.33 ± 1.02	22.13 ± 1.17

The contents of compounds have been calculated to percentage based on the dry weight of rice husk and rice straw. Data were collected and calculated from triplicate analyses

### Enzymatic saccharification of pre-treated rice husk and rice straw

**Fig. 1:**
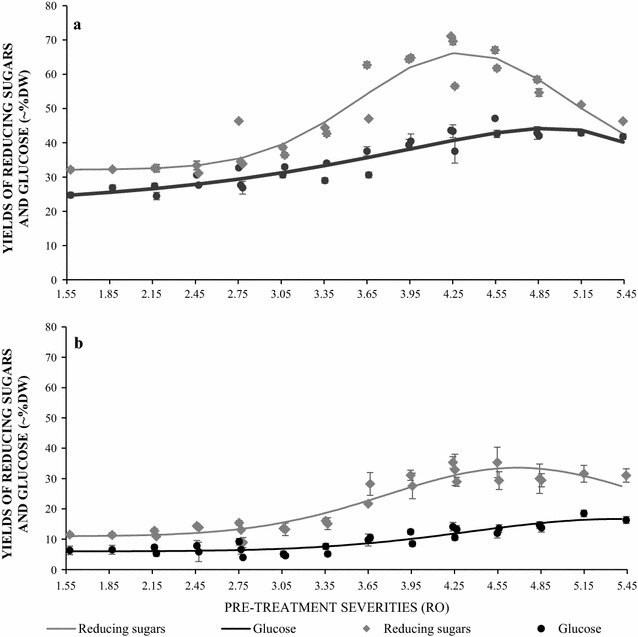
Saccharification of pre-treated rice straw and husk. Yields of both glucose and reducing sugars from rice straw (**a**) and rice husk (**b**) pre-treated at different severities, after a 96 h saccharification using cellulase (CTec-2) at 50 °C. The light grey curve represents the proportion of total reducing sugars in air-dried material and dark grey curve represents the proportion of glucose in air-dried material. *N* = 2; data were processed by using Genstart (Edition 18) to show the trend

Enzymatic saccharification of hydrothermally pre-treated rice husk and rice straw was performed in 15 ml volumes (5% w/v substrate) at 50 °C for 96 h. The results in Fig. 1 present the reducing sugar and free glucose yields as a function of pre-treatment severities. Overall, reducing sugar and glucose both increased with increasing severity. Consistent with the results of steam explosion, [19] enzymatic hydrolysis of hydrothermally pre-treated rice straw released much higher quantities of reducing sugars (maximum 66.1% at severity 4.27) and glucose (maximum 43.6% at severity 5.15) compared with rice husk (maximum 35.3% reducing sugar at severity 4.55 sugar and 16.3% glucose at severity 5.44). In rice husk, reducing sugar yield grew steadily with increasing severity up to 4.5 then slowly decreased, whilst glucose yield continued to increase at above this severity. In rice straw, reducing sugar yield reached a peak at a severity of 4.3 and then decreased rapidly at higher pre-treatment severities. In contrast to husk, the peak of glucose yield (at a severity of 4.8) was followed by a decrease in glucose yield at higher severities. Thus under similar conditions of pre-treatment and enzyme loading, significantly higher sugar and glucose yields were achieved from rice straw compared with rice husk.

### Simultaneous saccharification and fermentations (SSF)

SSF was carried out at a lower temperature (25 °C) by simultaneously adding cellulase (Ctec-2) and a yeast strain (*Saccharomyces cerevisiae NCYC 2826*) which ferments hexose sugars, but not pentoses [23]. Four pre-treatment severities spanning the range used above were selected from low to very high (1.57, 3.65, 5.35, and 5.45). The results (Table 2) show that: (1) ethanol yields were significantly higher from RS compared with RH after pre-treatment at severities 1.57 and 3.65, indicating that yeast behaves differently on the different lignocellulose hydrolysates; (2) ethanol yields were very low in both RH and RS pre-treated at severities 5.15 and 5.45, which suggests that yeast behaviour was being suppressed. Previously [23], we showed that washing pre-treated (steam exploded) rice straw prior to SSF reduced such severity-related decline in SSF efficiency and concluded that this was due to the removal of fermentation inhibitors. The impact of these inhibitors appears to be predominantly on the fermentation step as indicated by the data in Fig. 1 which shows that the saccharification of total pre-treated slurries occurs at all the severities.

**Table 2:** Ethanol produced from rice husk and rice straw pre-treated at four different severities (5% w/w of substrates in 15 ml slurry)

Severity (Ro)	Rice husk (~ %DW)	Rice straw (~ %DW)
1.57	3.57 ± 0.44	7.43 ± 1.27
3.65	7.55 ± 1.00	14.22 ± 2.08
5.15	1.07 ± 0.20	1.30 ± 0.51
5.45	1.31 ± 0.29	1.35 ± 0.19

Pre-treated samples were hydrolysed by Cetc-2 and fermented by *Saccharomyces cerevisiae* (NCYC 2826). Duplicates were carried out for ethanol quantification. Results were calculated as the proportion of dry weight of substrate (~ %DW)

### Chemical analysis of supernatants from pre-treated RH and RS by using nuclear magnetic resonance (NMR)

**Fig. 2:**
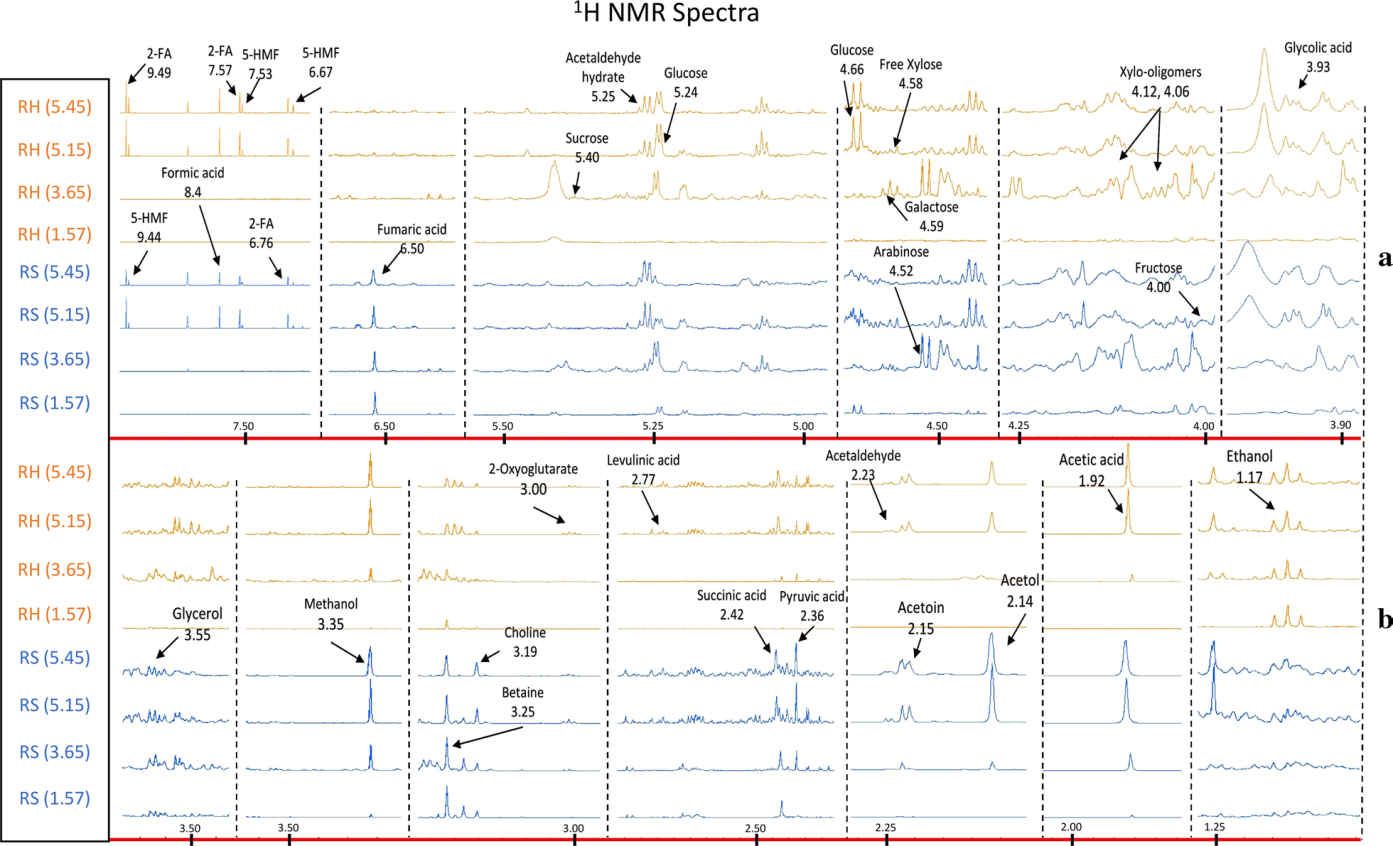
^1^H NMR spectra of 25 chemical compounds identified from the liquors of pre-treated rice husk and rice straw. Four severities (severities 1.57, 3.65, 5.15, 5.45) were selected as examples to present the identification method. The complete spectra were split into two main parts (**a**, **b**), which were further divided into several fragments and scaled differently to indicate compounds produced at low level. The red lines show the chemical shift (-ppm) scale with chemical shifts of individual compounds indicated on the figure

**Fig. 3:**
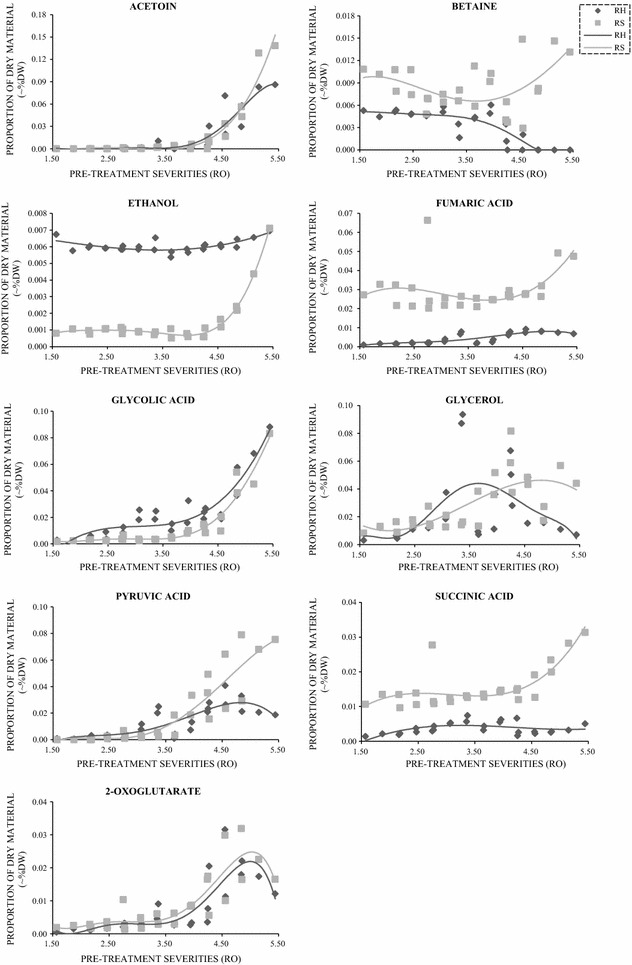
The trends of insignificant or non-inhibitory compounds detected in PTRH and PTRS. Compounds are presented as dry weight of the original substrate (%DW). Light grey: rice straw. Dark grey: rice husk

**Fig. 4:**
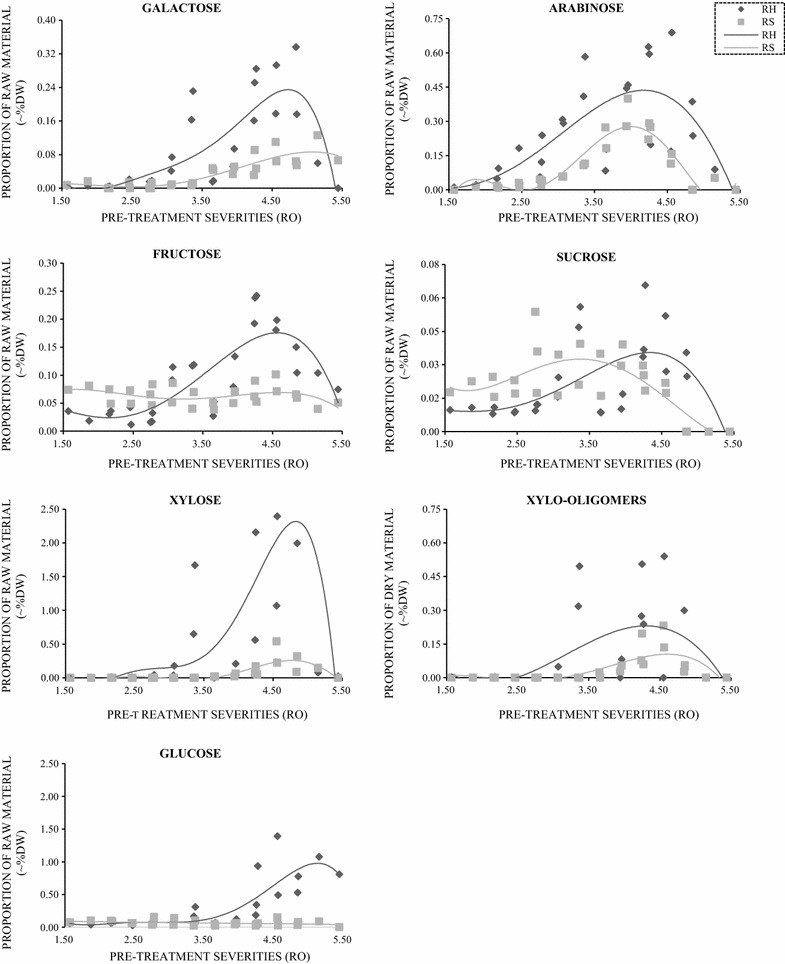
Sugars released during pre-treatment of rice straw and rice husk. Compounds are presented as dry weight of the original substrate (%DW). Light grey: rice straw. Dark grey: rice husk

**Fig. 5:**
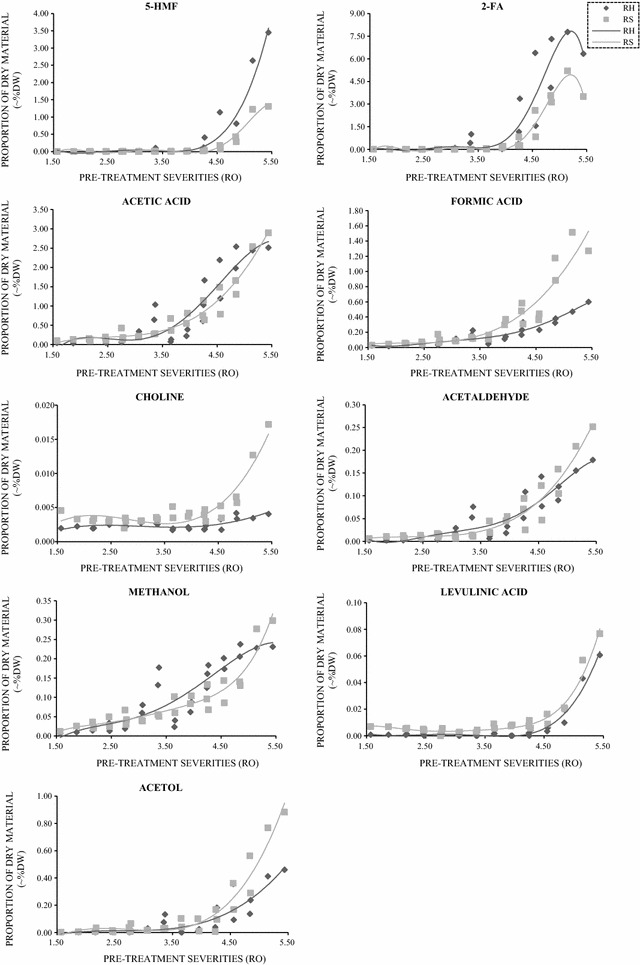
Fermentation inhibitors produced during pre-treatment of rice straw and husk. Compounds are presented as dry weight of the original substrate (%DW). Light grey: rice straw. Dark grey: rice husk

A more comprehensive understanding of the range of breakdown and solubilised components created during pre-treatment of the RH and RS was achieved by analysing the liquors by NMR. The results showed that 25 different compounds were readily detectable and quantifiable. The diagnostic spectral regions of the compounds for RH and RS samples pre-treated at severities 1.57, 3.65, 5.15 and 5.45 are shown in Fig. 2 (see Additional file 1: Figure S1 for a higher magnification version of the spectra), scaled to address variation in concentration. The quantities of these compounds, as affected by severity of pre-treatment are shown graphically in Figs. 3, 4 and 5. Acetaldehyde and acetaldehyde hydrate were quantified as one compound.

**Fig. 6:**
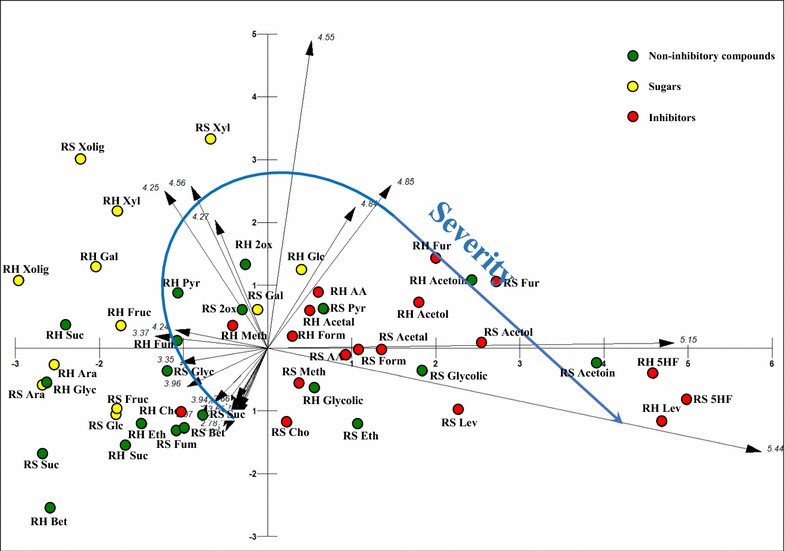
Principal components analysis. Distributions of 25 detected compounds (green, yellow and red points) released during hydrothermal pre-treatment of rice straw and rice husk in relation to severity of pre-treatment (arrow vectors)

Associations of those compounds with severities and with each other have been presented with principal component analysis (PCA) and shown in Fig. 6. Severities are shown by the vectors (arrows), whilst the chemical compounds released are shown as coloured circles. The components identified were categorised as: nine previously unidentified compounds (green circles, mostly positioned around low severity vectors, bottom left); 7 sugars (shown as yellow circles, positioned adjacent to moderate severity vectors); and 9 established fermentation inhibitors (shown as red circles, generally positioned to the right-hand side of Fig. 6 associated with the higher severity pre-treatment).

Figure 3 shows compounds created and/or released during low severity pre-treatments. Several of these are organic acids typically found in intermediary metabolism, namely pyruvic, succinic, fumaric and 2-oxoglutaric acids. In addition, acetoin, glycolic acid and glycerol were detected. Succinate, fumarate and pyruvate were produced in higher quantities at higher severities, and particularly in PTRS. Acetoin and glycolic acid increased consistently from low severities to high severities, but glycerol, pyruvic and 2-oxoglutarate started to decrease after reaching their peaks indicating degradation. Ethanol was produced in small quantities from both PTRH and PTRS (at higher severities). Betaine levels and trends differed between PTRH and PTRS, showing marked degradation at higher severities in PTRH.

At moderate pre-treatment severities, sugars and oligosaccharides were released (Fig. 4). These all showed similar trends in that the levels peaked at around a severity of 4.5 after which they decreased, presumably due to degradation (concomitant with the increase in fermentation inhibitors shown in Fig. 5). Generally, rice husk released higher amounts of sugar compounds than rice straw at any given severity. The presence of galactose may reflect the hydrolysis of small quantities of pectic polymers in the cereal biomass, whilst the xylose, xylo-oligomers and arabinose are likely to be derived from xylans and arabinoxylan hemicelluloses.

Compounds known to cause significant inhibition on saccharification or fermentation were released at higher severities and are shown in Fig. 5. In keeping with previous studies [19], most of the inhibitors increased with increasing severities. Hydroxy-methyl furfural (5-HMF), furfural (2-FA) and acetic acid were the most abundant inhibitors produced from both PTRH and PTRS. Complementing Wood et al. [19], considerably higher levels of all the inhibitors were produced from rice husk at the higher severities, consistent with the higher levels of sugar release and breakdown shown in Fig. 4. However in the present study, the levels of 5-HMF, 2-FA and acetic acid produced at the much higher severities were very much greater than those reported by Wood et al. [19] (confirmed by HPLC—results not shown). This may be due to two factors: firstly, in the previous study, the maximum pre-treatment severity was 4.8, whilst in this study the severity went to higher levels; secondly, it is very likely that considerable quantities of these volatile compounds were lost into the vented steam during the explosion process. Measurable amounts of formic acid, acetol, acetaldehyde and methanol were also produced significantly from pre-treated samples. Choline and levulinic acid were produced at much lower levels than the other inhibitors and were produced more from PTRS than PTRH.

## Discussion

Rice straw and husk are important sources of biomass globally and have similar chemical compositions. However, after identical hydrothermal pre-treatments, rice husk is poorly saccharifiable, produces higher levels of fermentation inhibitors and yields much less ethanol during SSF. There are several factors that may be responsible for the higher recalcitrance. Firstly, the level of lignin is considerably higher in rice husk. This will not only act as a dense, physical barrier to enzymolysis [24], but will also provide a larger physical surface onto which cellulases may bind strongly [25], reducing the availability of free enzyme. Secondly, larger quantities of xylose and xylo-oligosaccharides are released during pre-treatment of rice husk compared with those released from rice straw (Fig. 4) at concentrations calculated to be in the order of over 1 mg/ml. Such concentrations have been shown to severely inhibit cellulase activity [26]. Thirdly, whilst not assessed in this study, rice straw and rice husk contain considerably higher levels of silica in their cell walls compared with other cereal lignocellulose, and it is much higher in rice husk than in rice straw [27]. Silica has a severe impact on ruminant digestibility of rice straw and husk [27] and would therefore be expected to have an impact on the digestibility of pre-treated rice biomass. Many researchers have shown that it is possible to use very harsh chemicals to overcome rice straw and husk recalcitrance by extracting lignin and other structural barriers to enzymolysis [19]. For example, Ang et al. [28] compared a range of additions of chemicals and reached 22.3% (w/w) yield of total sugar after pre-treating rice husk by adding HCl without any further hydrolysis. Saha and Cotta [29] used alkaline peroxide to achieve a saccharification yield of 42.8% (w/w). Recently, Khaleghian et al. [30] demonstrated that the chemical removal of silica after previously removing lignin considerably enhances saccharification. However, such treatments generally employ very large quantities of chemicals, often of the same order of magnitude as the biomass being treated. This will be costly both financially and environmentally [31, 32]. It is clear that further studies on the role of silica in recalcitrance are required.

The in-depth study of pre-treatment liquors by NMR has also shown that hydrothermal (hot water) pre-treatments retain large amounts of commonly known fermentation inhibitors and a range of other compounds. These have a highly deleterious impact on fermentation [33, 34]. Whilst they could be removed by washing the substrate [19], this would add a further processing step and would remove much of the solubilised sugars which may also be exploited in a single processing step using modified fermenting organisms that can ferment pentoses effectively. However, we have also suggested that some might be substantially removed by volatilisation during steam explosion. Some moieties have varying functionality. For example at low severities, acetaldehyde was produced at concentrations (0.01 mg/ml) that can enhance fermentation through reducing the lag phase of yeast growth [35], whilst at higher severities acetaldehyde was produced at concentrations (over 0.1 mg/ml) where it can inhibit yeast growth [36]. Metabolites and organic acids produced in small quantities at low severities are not generally recognised. Such moieties could act as substrates for the fermenting microorganism.

Whilst the development of economically viable pre-treatments for rice husk remains a considerable challenge, it may be possible to develop a tractable processing regime for rice straw. In this study, RS samples pre-treated between severities of 3.65 and 4.25 would give a promising yield of glucose which is circa 10% lower than the maximum yield (Fig. 1). Also, at the severity of 3.65, the levels of inhibitors are on the lower end of the range and their volatilisation by steam explosion may reduce them further. Future exploitation of rice husk may require targeted breeding strategies to address the recalcitrant properties.

## Conclusion

Rice straw and husk are important global sources of biomass for biorefining. Rice husk presents a much greater degree of post-hydrothermal pre-treatment recalcitrance compared with rice straw due, probably, to high levels of lignin and silica. It also produces higher levels of fermentation inhibitors during hydrothermal pre-treatment. In-depth analysis of the pre-treatment liquors by NMR has identified a wide range of components created throughout the severity range. At low severities, metabolites including organic acids are extracted—these are generally broken down at severities above 4. At mid-range severities, a range of sugars and oligosaccharides are released presumably through hydrolysis of cell wall polysaccharides; many of these are lost at the highest severities where there is a rapid increase in well-established fermentation inhibitors. It is postulated that these might be reduced by volatilisation through steam explosion, rather than adding additional washing steps that would lose many potentially fermentable components.

## Materials and methodology

### Raw materials

Rice husk and straw were provided as described previously [19].

### Milling substrates

Air-dried rice husk and straw (200 g) were chopped with scissors into about 2 cm lengths and then micronised using a RETSCH cyclone mill (Retsch Limited, Hope Valley, United Kingdom) with a 0.5 mm mesh. The milled material was recovered into screw-capped sample pots and stored under laboratory conditions. Rice husk and straw (< 0.5 mm) were firstly pre-frozen using liquid nitrogen for 10 min. and then further milled using a 6700EFM Freezer/Mil (SPEX Sample Prep, Stanmore, UK).

### Sugar analysis of air-dried rice husk and straw

Milled rice husk and straw were hydrolysed and saccharified by using 72% (w/w) H_2_SO_4_ at room temperature for 3 h followed by 1 mol/l H_2_SO_4_ at 100 °C for 2.5 h after Saeman [37]. Hydrolysed samples were reduced using sodium borohydride (NaBH_4_) and acetylated by addition of 1-methylimidazole and acetic anhydride by the method described Blakeney et al. [38]. Gas chromatography (Perkin Elmer Autosystem XL, Perkin Elmer, Seer Green, UK) and a RTX-225 column (Restek, Bellefonte, USA) was used to analyse the alditol acetates produced from the monosaccharides. This had been carried out three times.

### Lignin analysis of rice husk and straw

Sintered glass funnels (porosity 4) (VWR International Ltd, 1151 Budapest, Szövőgyár utca 11–13, Hungary) were placed in an oven to remove moisture (50–60 °C) for obtaining the weight of each funnel (WT funnels). Milled rice husk and rice straw (100 mg) were loaded into Sovirel tubes (The Science Company, 7625 W Hampden Ave, Unit 14, Lakewood, Colorado, US) and hydrolysed by adding 1.5 ml of 72% sulphuric acid and then incubated at 25 °C for 3 h. After the first incubation, 18 ml distilled water was added into each tube and further incubated at 100 °C for 2.5 h. Hydrolysates of rice husk and rice straw were then filtered with sintered glass funnels and washed with distilled water for removing acids. Funnels containing hydrolysates were dried at 50 °C until a constant weight was obtained and the weight of each funnel was recorded (WT funnels + WT hydrolysates). Dried funnels containing hydrolysates were then placed into a Vulcan PD Furnace 3-550 (Dentsply Sirona Global Headquarters, Susquehanna Commerce Centre. 221 West Philadelphia Street, Suite 60 W, York PA, US) at 500 °C for 22 h for obtaining ash weight (WT funnels + WT ash). Samples in this experiment had been prepared in triplicate. Weight of lignin was calculated as follows:

$$\begin{aligned} {\text{Lignin}} & {\text{ = WT funnels + WT hydrolysates}} \\ & \quad - {\text{(WT funnels + WT ash) [mg/g Raw materials]}}. \\ \end{aligned}$$

### Pre-treatment of milled rice husk and straw

Rice husk and straw were pre-treated by using a BIOTAGE^®^ Initiator + reactor (Biotage AB, Box 8, 751 03, Uppsala, Sweden). Milled husks and straw (0.75 mg for each tube) had been added separately into 25 of 20 ml microwave pressure tubes (containing 14.25 ml distilled water individually) to give a 5% (w/w) suspension. The tubes were then capped and treated at pre-designed pre-treatment severities. Pre-treatment severity was calculated from temperature and duration using the following equation (adapted from Overend et al. [39]):

$${\text{Severity }}(Ro) = \log_{10} \left(t \cdot exp^{{\frac{T - 100}{14.75}}} \right).$$

The range of severities as a function of time and temperature is shown in Table 3 (conditions of empty cells had not been tested because their severities were already provided by other conditions). Those tubes were frozen before further experiment after tubes were cooled with compressed air to room temperature.

**Table 3:** Pre-treatment severities

Time (min)	Pre-treatment severities							
	Temp (°C)							
	140	150	160	170	180	190	200	210
2.5	*1.57*	1.87	2.16	2.46	2.75	\	\	\
10	2.18	2.47	2.77	3.06	3.35	*3.65*	3.94	4.24
40	2.78	3.07	3.37	3.66	3.96	4.25	4.55	4.84
160	\	\	\	4.27	4.56	4.85	*5.15*	*5.44*

Italic values indicate severities used during the SSF studies

### Saccharification of pre-treated rice husk and straw

Pre-treated samples were defrosted fully and then 5 ml of buffer (0.4 M sodium acetate acetic acid buffer, pH 5.0) containing 0.04% v/v thimerosal was added. Cellic^®^ CTec-2 (Novozymes, Denmark) (187.5 µl) was then added into samples. After those samples had been finally capped, they were incubated (120 rpm) at 50 °C for 96 h. The content of glucose and total sugars of hydrolysates were analysed by GOPOD Format (d-glucose assay kit, Megazyme, US) and DNS (dinitrosalicylic acid method) adapted method reported by Wood et al. [40], respectively. Duplicates were prepared in this experiment.

### Simultaneous saccharification and fermentations (SSF)

SSF was investigated using 1 ml Matrix Tubes (Thermo Fisher Scientific, Waltham, MA USA) with rice straw and husk pre-treated at four severities (pre-treatment conditions italicised in Table 3). Suspensions of pre-treated slurries were stirred rapidly to enable quantitative transfer of 937.5 µl into Matrix tubes. After the addition of Cellic^®^ CTec2 (Novozymes, Denmark) (12.5 µl) and pre-grown yeast strain (*Saccharomyces cerevisiae NCYC 2826,* 50 µl) to each Matrix tube, they were sealed with screw caps and set into Matrix plates. Capped Matrix plates were placed on shaker (135 rpm) under 25 °C for 72 h. They were then heated at 100 °C for 10 min to deactivate enzyme and yeast. After cooling with ice and centrifuging (3000 rpm) for 10 min, 400 µl supernatants of each sample was filtered using 0.2 µm filter plates (Pall Corporation, World Headquarters, Washington USA) and then transferred into a 96 well deep-well (1 ml round bottom) plate for HPLC analysis. Ethanol standards were made for quantifying ethanol products from yeast fermentation.

### Chemical analysis of liquors from pre-treated rice husk and rice straw

^1^H nuclear magnetic resonance (^1^H NMR) was used to identify the presence and concentration of compounds released and generated from raw materials during pre-treatment. A phosphate buffer was generated by combining NaH_2_PO_4_·H_2_O (8.4 g), K_2_HPO_4_ (3.3 g), NaN_3_ (40 mg), and sodium 3-(trimethylsilyl)-propionate-*d*_*4*_ (TSP, 17.2 mg) with 200 ml D_2_O. The liquors of pre-treated samples were thawed at room temperature and prepared for ^1^H NMR spectroscopy by mixing 400 µl of spent medium with 400 µl of phosphate buffer. The sample was mixed, and 500 µl was transferred into a 5-mm NMR tube for spectral acquisition. The ^1^H NMR spectra were recorded at 600 MHz on a Bruker Avance spectrometer (Bruker BioSpin GmbH, Rheinstetten, Germany) running Topspin 3.2 software and fitted with a cryoprobe and a 60-slot autosampler. Each ^1^H NMR spectrum was acquired with 64 scans, a spectral width of 12,500 Hz and an acquisition time of 2.62 s. The “noesygppr1d” pre-saturation sequence was used to suppress the residual water signal with a low-power selective irradiation at the water frequency during the recycle delay. Spectra were transformed with a 0.3-Hz line broadening, manually phased, baseline corrected and referenced by setting the TSP methyl signal to 0 ppm. Absolute concentrations were obtained by using CHENOMX software (version 5.1) supplemented by in-house additions to the CHENOMX compound library, with quantification calculated relative to TSP.

### Principal components analysis (PCA)

PCA was carried out using Multi Variate Statistical Package version 3.22, Kovach Computing Services, Anglesey, UK.

## Additional file

**Additional file 1: Figure S1.** Magnified version of Fig. 2: ^1^H NMR spectra of 25 chemical compounds identified from the liquors of pre-treated rice husk and rice straw. Four severities (severities 1.57, 3.65, 5.15, 5.45) were selected as examples to present the identification method. The complete spectra were split into two main parts (A and B) which were further divided into several fragments and scaled differently to indicate compounds produced at low level. The red lines show the chemical shift (-ppm) scale with chemical shifts of individual compounds indicated on the figure.

## Abbreviations

5-HMF: hydroxymethyl furfural
2-FA: furfural
SSF: simultaneous saccharification and fermentation
NCYC: National Collection of Yeast Cultures
NMR: nuclear magnetic resonance
PCA: principal component analysis
HPLC: high-performance liquid chromatography
TSP: sodium 3-(trimethylsilyl)-propionate-d4
GOPOD: d-glucose assay kit
DNS: dinitrosalicylic acid method
