# Supplementary figures and images for: Optimising conditions for bioethanol production from rice husk and rice straw: effects of pre-treatment on liquor composition and fermentation inhibitors
Source: Biotechnol Biofuels. 2018 Mar 9;11:62. doi: 10.1186/s13068-018-1062-7 (PMC5844111; doi:10.1186/s13068-018-1062-7)

# $^1\text{H}$ NMR Spectra

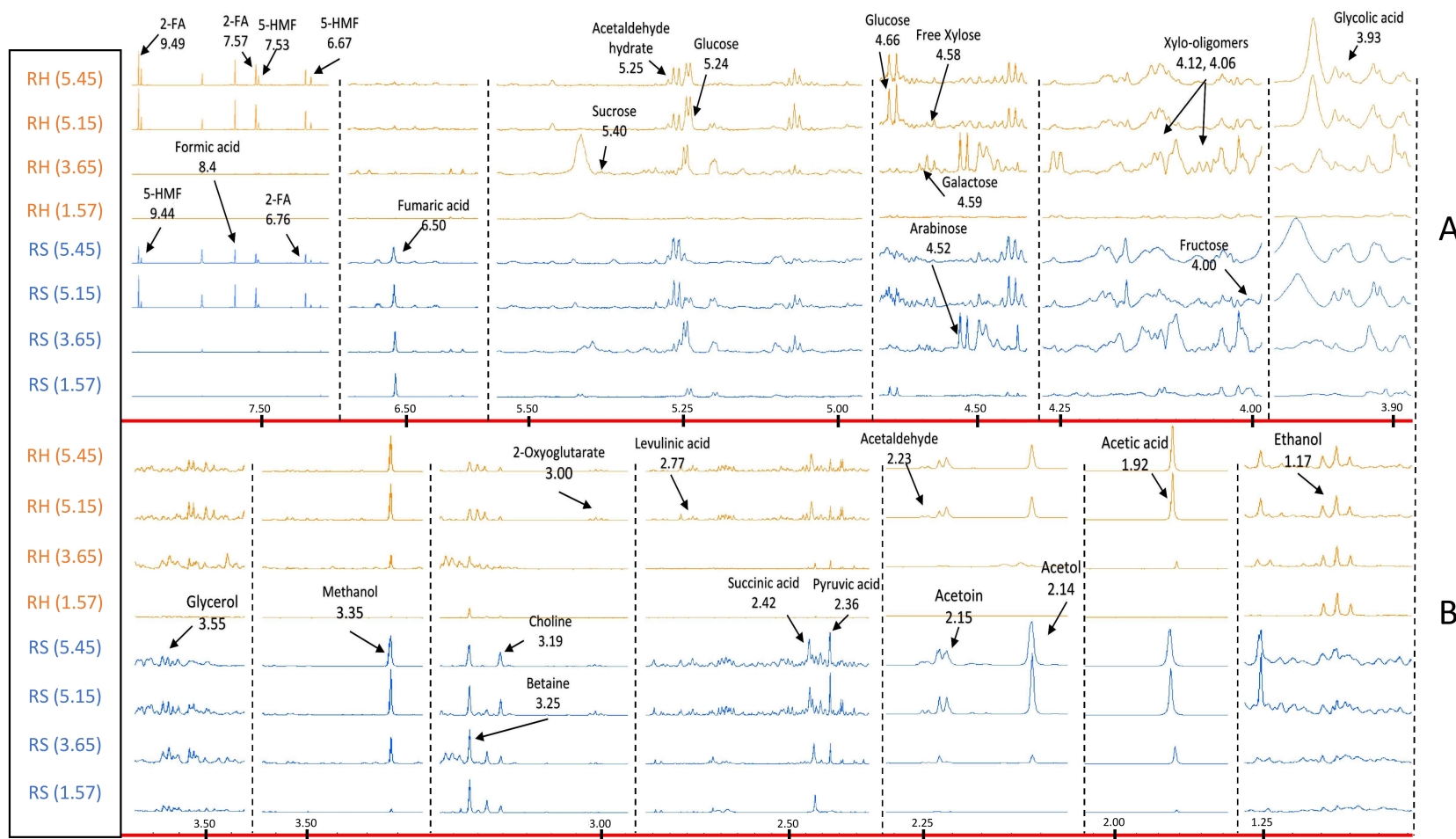

Supplement: Supplementary file 1 — Additional file 1: Figure S1. Magnified version of Fig. 2: 1H NMR spectra of 25 chemical compounds identified from the liquors of pre-treated rice husk and rice straw. Four severities (severities 1.57, 3.65, 5.15, 5.45) were selected as examples to present the identification method. The complete spectra were split into two main parts (A and B) which were further divided into several fragments and scaled differently to indicate compounds produced at low level. The red lines show the chemical shift (-ppm) scale with chemical shifts of individual compounds indicated on the figure. [file 13068_2018_1062_MOESM1_ESM.pdf]
